# Incidental Petrous Apex Cephalocele Presenting With Transient Global Amnesia: A Case Report and Rapid Literature Review

**DOI:** 10.7759/cureus.51778

**Published:** 2024-01-07

**Authors:** Alexandros Brotis, Mariana Vlychou, Ioannis Ioannidis

**Affiliations:** 1 Neurosurgery, General University Hospital of Larissa, Larissa, GRC; 2 Radiology, Medical School of Thessaly, Larissa, GRC

**Keywords:** asymptomatic, magnetic resonance imaging, empty sella, transient global amnesia, petrous apex cephalocele

## Abstract

Transient global amnesia (TGA) constitutes a rare clinical entity that manifests with temporary memory without any other neurological manifestation. Several pathogenetic mechanisms have been implicated, including temporal hypoperfusion, venous congestion, and cortical spreading potentials. Accordingly, the only relevant imaging findings are hippocampal CA1 areas of restricted diffusion on diffusion-weighted images. In the current case report, we present the rare case of a patient with TGA associated with bilateral petrous apex cephalocele (PAC).

A 63-year-old female presented with a single episode of transient memory. The brain MRI showed a bilateral PAC and an empty sella. The patient was neurologically intact upon examination and was conservatively managed. There was no symptom recurrence during the six months of follow-up. We hypothesize that the presence of the meningocele could be associated with the pathogenesis of TGA.

To the best of our knowledge, this is the first case of a petrous apex meningocele presenting with TGA. Most previously reported patients were females in their fourth decade of life, usually presenting with headaches or incidentally. Almost half of the cases were bilateral, with an empty sella. Surgical treatment was reserved for symptomatic patients with cerebrospinal fluid leaks and excruciating trigeminal neuralgia.

Patients with TGA may be associated with temporal lesions, including PAC. Likewise, PAC is an extremely rare clinical entity that could occasionally present with TGA.

## Introduction

Transient global amnesia (TGA) is characterized by a sudden, complete memory loss lasting several hours without any other neurological manifestation [1]. The patient frequently repeats stereotype questions at irregular intervals [1]. The initial case description was made by Bender in 1956, while Fisher and Adams first used the term TGA in 1964 [2,3]. Currently, the diagnosis is usually based on the Hodges and Warlow criteria, which include an episode of witnessed amnesia without any other disorder of higher cortical function and cannot be attributed to a transient ischemic attack or epilepsy [4-7]. It occurs with a reported incidence as high as three to eight cases per 100,000 population, commonly affecting middle-aged individuals, and it is frequently associated with significant secondary psychological distress [1,7].

The underlying pathophysiological mechanisms are largely unknown, but several theories involving temporal hypoperfusion, venous congestion, and cortical spreading potentials have been implicated [1,7-9]. In cases of recurrent TGA, brain MRI helps rule out a stroke [7,10-12]. Brain imaging with head CT and MRI usually shows no abnormality [7]. Studies with a few patients identified hippocampal CA1 areas of restricted diffusion on diffusion-weighted images (DWI) [7,13-16]. 

Herein, we present the case of a patient with TGA associated with a petrous apex cephalocele (PAC). The relevant clinical and radiological characteristics are discussed, along with a review of the pertinent literature.

## Case presentation

A 63-year-old female presented at the outpatient department of our hospital complaining of an episode of transient memory loss and confusion lasting for about six hours. The patient was in her working environment, and witnesses reported that she was disoriented in time and space and could not recognize her colleagues. The patient was unaware of her current position and could not recall previous actions. She repeatedly asked questions about what had happened and where she was. The episode was not preceded by a recent head trauma or was associated with involuntary body and face movements.

Upon clinical examination, the episode was over, and the patient could not recall it. No other neurological findings occurred from the general mental status, cranial nerves, long tracts, cerebellum, and basal ganglia. The patient reported no previous memory loss events from her past medical history, and she received oral medications for hypertension and diabetes mellitus. The patient reported no smoking, alcohol, or recreational drug use. All laboratory examinations were within normal ranges, including the complete blood count, basic metabolic panel, and toxicology.

**Figure 1:**
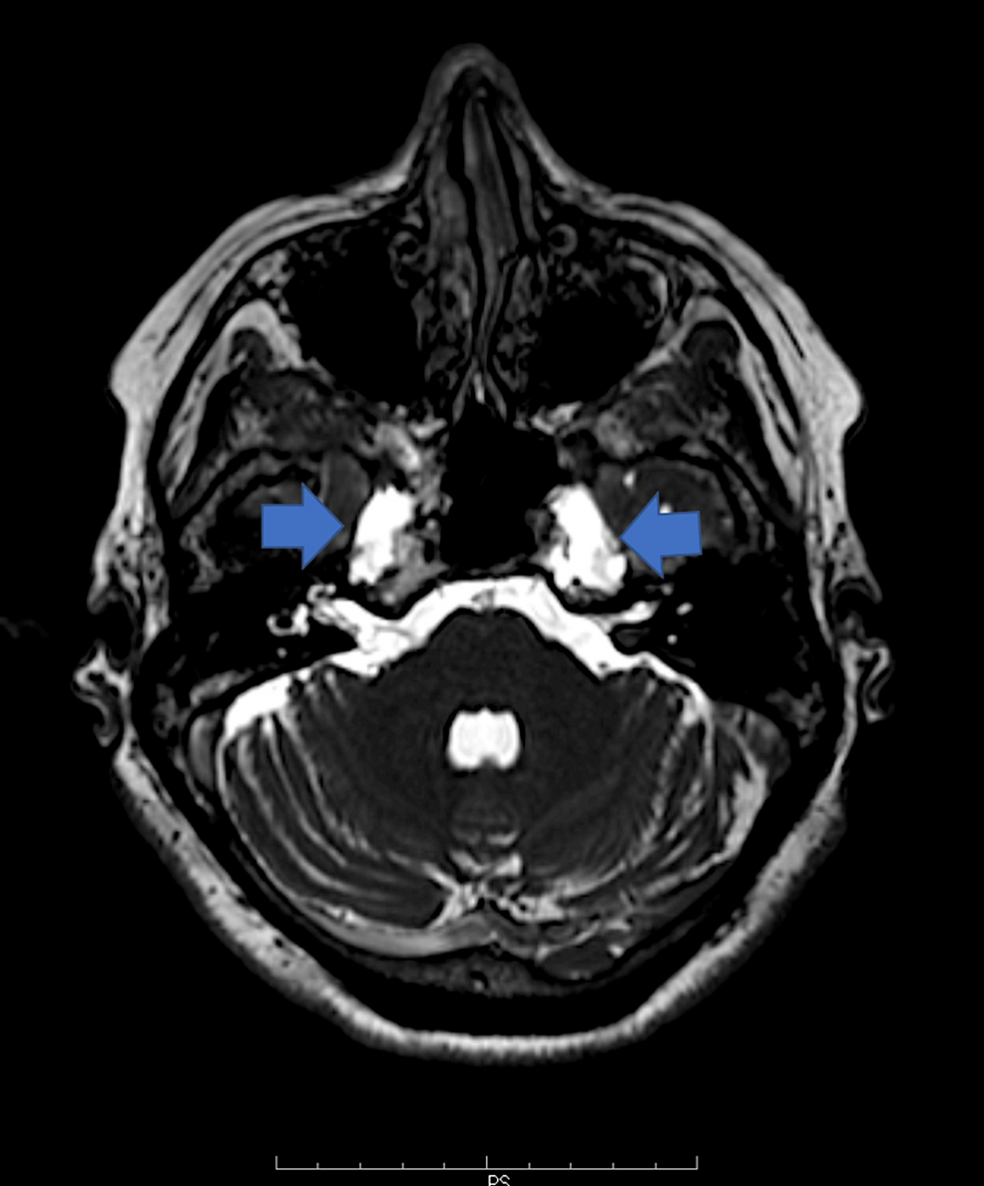
Axial T2 weighted images at the level of the facial nerve of a 63-year-old patient with TGA and bilateral PAC The blue arrows show the PAC

**Figure 2:**
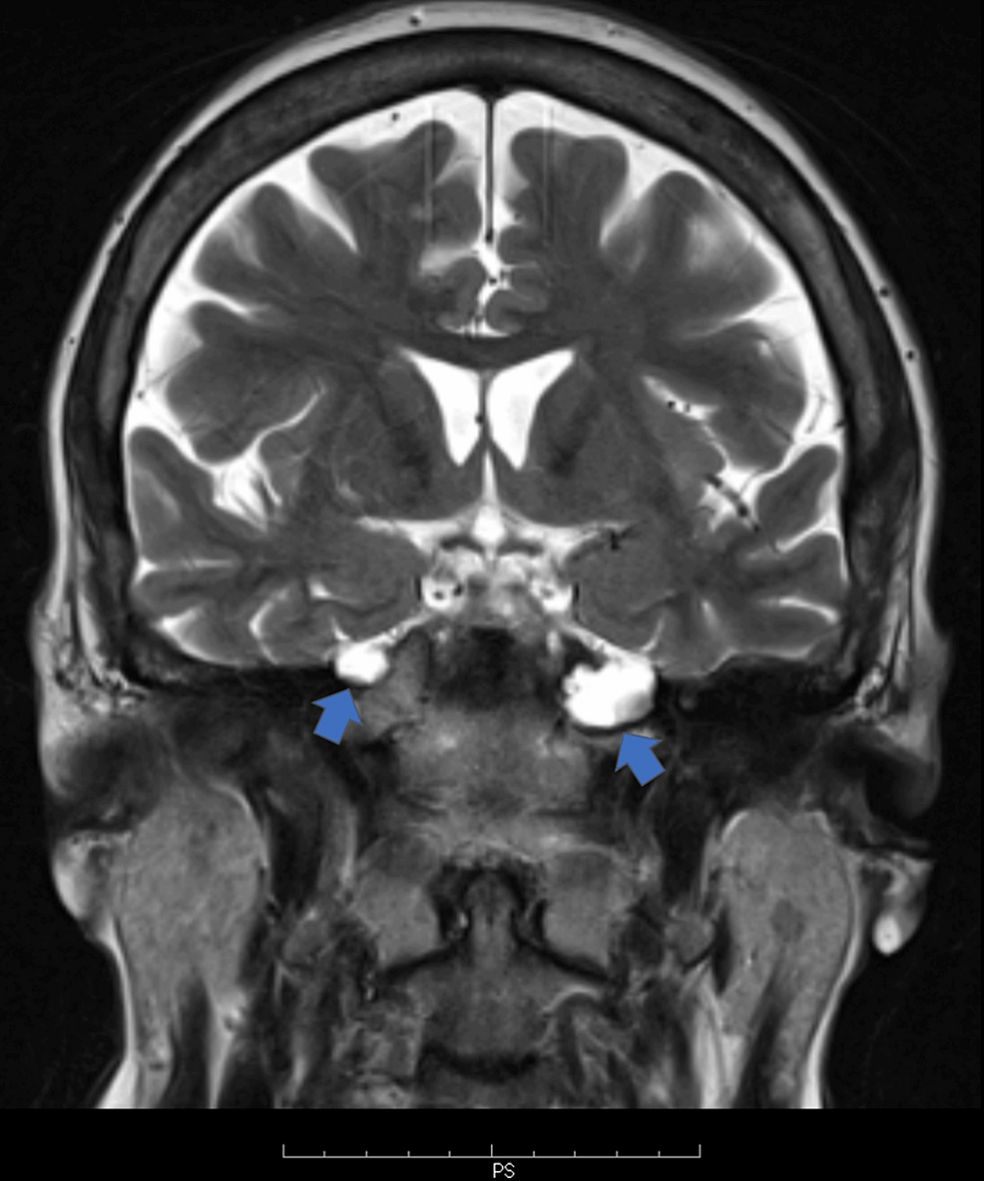
Coronal T2 weighted images at a level behind the anterior communicating artery of a 63-year-old patient with TGA and bilateral PAC The blue arrows show the PAC

**Figure 3:**
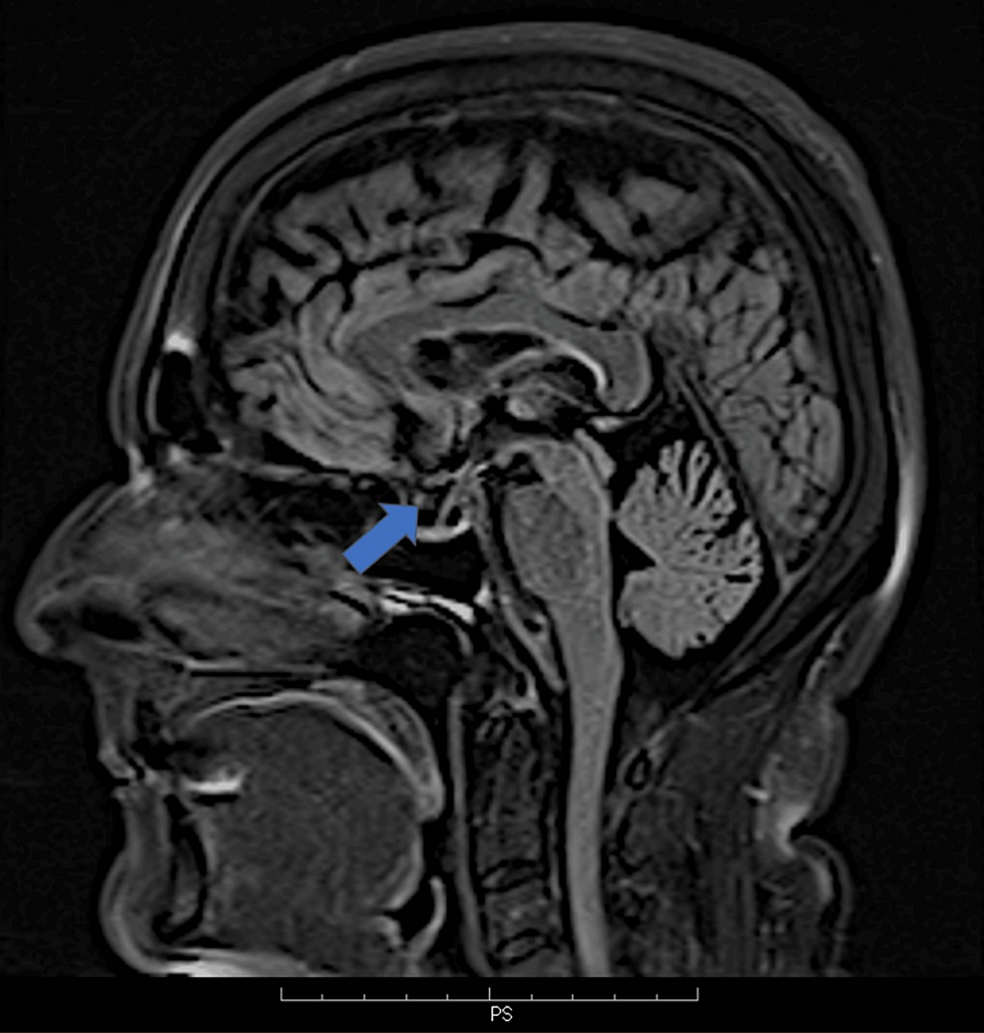
Sagittal FLAIR images of a 63-year-old patient with TGA and bilateral PAC The blue arrows show the compressed pituitary gland and its pituitary stalk

On MRI, we identified a bilateral (more prominent on the left), asymmetric, cystic-appearing lobular lesion at the petrous apices. The lesions extended from Meckel’s caves into the petrous apices with a CSF signal intensity on all pulse sequences, including a low signal intensity on T1W and fluid-attenuated inversion recovery (FLAIR) images and a high signal intensity on T2W images, and without signal restriction in the DWI sequence (Figures 1-3). An associated asymptomatic empty sella with a pituitary height of 2 mm (grade III) was also noted. The sella turcica was filled with CSF, the pituitary stalk was clearly visible, and the pituitary gland was compressed to the sellar floor. There were no findings consistent with TGA, including small punctuate lesions of restricted diffusion on DWI images in the CA1 area of the hippocampus.

Based on the patient’s history, clinical examination, and laboratory findings, a diagnosis of TGA was made. Since the patient had improved by the time of the examination without any intervention, we initiated no further treatment. Instead, the patient was reassured and advised to visit the emergency department should another episode occur. The patient was followed up for about six months and did not complain of recurrent amnesia episodes or any other neurological manifestation.

Material and methods

We performed a literature search and a rapid literature review. Thus, we searched PubMed using as keywords the terms “petrous apex cephalocele” OR “cavum trigeminale cephalocele” OR “Meckel’s cave cephalocele” in PubMed. We excluded articles written in languages other than English. The gathered articles were screened based on the title's relevance and abstract. The full text of the relevant articles was retrieved, and articles reporting on pathologies other than the pathology under study were eliminated. The remaining articles were summarized and formed the basis of our rapid review. We did not assess the reporting quality in anticipation of underpowered and low-quality studies.

Literature review

Our literature search identified 26 articles with 208 cases of PAC, written between 1997 and 2022 (Table 1). Cephaloceles are usually defined as meningeal diverticula with CSF, known as meningoceles, or may also contain part of the brain, defined as encephaloceles [17,18]. PAC occurred at any age from 1.5 to 86 years, but the majority affected patients in the sixth and seventh decades of life. Females (n=124, 59.6%) were almost twice as commonly affected as males. More than half of the patients (n=119, 57%) hosted bilateral lesions. In most cases, the recognition of the PAC was incidental and occurred during the diagnostic workup for recurrent headaches and other non-related findings [19-26]. On the contrary, symptomatic cases manifest with trigeminal neuralgia, CSF leaks with or without meningitis, and symptoms and signs of altered intracranial pressure. Notably, CSF leaks were frequently associated with meningitis [17,18,27-30]. The bibliography identified several heterogeneous clinical manifestations, such as cranial nerve palsies, hearing loss, and bilateral papilledema [17-19,22,30-40].

**Table 1:** The basic characteristics of the cases reported in the literature CSF: cerebrospinal fluid; NA: not available; TIA: transient ischemic attack; TN: trigeminal neuralgia

Author (year)	Number	Age (years)	Female (%)	Bilateral (%)	Empty sella (%)	Presentation	Other imaging findings	Management	Outcome
Mulcahy et al. (1997) [27]	3	6, 9, 25	1 (33)	0 (0)	0 (0)	Recurrent meningitis, partial complex seizures, headaches	NA	Surgical	Hemiparesis and hemianopsia
Moore et al. (2001) [18]	10	48 (range 5-82)	8 (10)	3 (30)	0 (0)	Headaches, CN palsy, TN, CSF leak, incidental	NA	Surgical	NA
Motojima et al. (2005) [28]	1	6	1 (100)	0 (0)	0 (0)	Recurrent meningitis	NA	Surgical	Complete recovery
Isaacson et al. (2006) [17]	4	85, 55, 44, 69	3 (75)	0 (0)	0 (0)	Right abducens palsy, vertigo, otalgia, diplopia, meningitis, hearing loss, retroorbital headaches	NA	Surgical	NA
Alorainy (2007) [19]	5	47 (range 25-60)	4 (80)	4 (80)	4 (80)	Incidental	NA	NA	NA
Stark et al. (2009) [39]	1	58	0 (0)	1 (100)	0 (00	Usher syndrome	Diffusely enlarged subarachnoid spaces surrounding the exits of cranial nerves (CN) II-XII	Conservative	Resolution
Hatipoğlu et al. (2010) [20]	4	48 (range 41-60)	4 (100)	4 (100)	3 (75)	Headache, diplopia	Ischemia, gliosis, atrophy, arachnoid cyst	NA	NA
Jeong et al. (2011) [29]	2	42, 66	2 (100)	2 (100)	1 (50)	Progressive bilateral tinnitus and mild hearing impairment	NA	Conservative	Amenorrhea, complete recovery
Jakkani et al. (2012) [21]	1	39	0 (0)	0 (0)	0 (0)	Recurrent headaches	NA	NA	NA
Alobaid et al. (2015) [32]	1	64	1 (100)	0 (0)	0 (0)	CSF leak and TN	NA	Surgical	Complete resolution
Boppel et al. (2015) [33]	5	48 years	4 (80)	3 (60)	2 (40)	Progressive hearing loss, progressive loss of vigilance, TIA	Bony erosions of the skull base	NA	NA
Çavusoglu et al. (2015) [34]	13	26-69	12 (92)	8 (61)	8 (61)	Headache, vertigo, CSF leak, TN	Intracranial aneurysmal dilatation, empty sella, mass in the hypophysis, arachnoid cyst, inferior herniation of parahippocampal gyrus, and optic nerve sheath CSF distension	NA	NA
Jamjoom and Alorainy (2015) [22]	111	6-81	87 (78)	77 (6)(	77 (69)	Headache, visual disturbances and papilledema, incidental, and TN	NA	NA	NA
Warade and Misra (2016) [29]	1	26	0 (0)	0 (0)	0 (0)	CSF leak	NA	Surgical	Complete recovery
O'Connell et al. (2016) [38]	1	56	1 (100)	1 (100)	1 (100)	Right ppulsatile tinnitus	Left superior semicircular canal dehiscence	NA	NA
Canan et al. (2017) [23]	1	61	1 (100)	1 (100)	0 (0)	Incidental	NA	NA	NA
Alkhaibary et al. (2020) [24]	1	64	1 (100)	1 (100)	1 (100)	Headache	Invasive erosions of the osseous structures	Conservative	NA
Gozgec and Ogul (2020) [42]	1	63	0 (0)	1 (100)	0 (0)	Headache and weakness	Coexisting meningioma	Surgical	NA
Epsten et al. (2021) [30]	35	55 (4-86)	29 (82)	9 (26)	13 (0.37)	Incidental, CSF leak, increased ICP, TN	Arachnoids pits	Surgical	NA
Erginoglu et al. (2021) [35]	1	56	1 (100)	0 (0)	0 (0)	TN	NA	Surgical	NA
Miccoli et al. (2021) [36]	1	1.5	0 (0)	0 (0)	1 (100)	Otitis media, CN VI palsy, bilateral papilledema	Bilateral ectasia of the optic nerve sheath	Surgical	Significant improvement
Pruthi et al. (2021 )[25]	1	75	1 (100)	1 (100)	1 (100)	Headache, neck pain, and lower back ache	NA	Conservative	Significant improvement
Dogan and Ozgur (2022) [26]	1	56	1 (100)	1 (100)	0 (0)	Non-specific headache	NA	NA	NA
Hanai et al. (2022) [44]	1	12	0 (0)	0 (0)	0 (0)	Chronic otitis media, orthostatic headache, nausea, visual loss	Enlarged superior ophthalmic and angular veins	Conservative	Complete resolution
Martinez et al. (2022) [37]	1	28	1 (100)	0 (0)	0 (0)	TN	NA	Surgical	Complete resolution
Truong et al. (2023) [40]	1	72	0 (0)	1 (100)	0(0)	Right-sided hearing loss and tinnitus	Bilateral fluid signal along the labyrinthine, geniculate, and proximal tympanic segment of the facial canal	Conservative	Stable
Current case (2023)	1	63	1 (100)	1 (100)	1 (100)	Transient global amnesia	NA	Conservative	Complete resolution

The diagnosis was usually set with brain CT and MRI scans showing a sharply circumscribed lucid expansile lesion of the petrous apex, continuous with Meckel’s cave, pointing posteriorly and inferiorly [19]. The most frequent coexisting radiological finding was an empty or a partially empty sella (n=113, 54%).

The management of incidental PAC follows the “leave-them-alone” strategy [41]. However, surgical exploration through an intradural or extradural approach is reserved for symptomatic cases with CSF leaks or devastating trigeminal neuralgia [17,27-30,32,35-37,41,42]. The final choice between the intradural and extradural approaches largely depends on inherent anatomical patient characteristics [43]. The postoperative outcome is usually excellent, followed by complete symptom resolution [28,29,31,32,36,37,44]. It is worth noting that the duration of the postoperative follow-up is vastly underreported.

## Discussion

We reported the case of a middle-aged female with an incidental PAC who was diagnosed after an episode of TGA. The clinical presentation was typical for TGA, and the radiological findings were specific for PAC. To the best of our knowledge, and after a thorough search in the relevant literature, this is the first PAC case associated with TGA.

The petrous apex is a pyramidal portion of the petrous part of the temporal bone [45,46]. It is bordered superiorly and inferiorly by the petrous ridge that contains the superior petrosal sinus and the petroclival suture overlaid with the inferior petrosal sinus [45,46]. Its base is formed by a virtual line extending from its anterior edge to the porus acusticus [45,46]. The internal carotid arteries and the bony labyrinth are located anteriorly, whereas the posterior cranial fossa starts just behind it [45,46]. Finally, Meckel's cave and the jugular bulb constitute the superior and inferior borders of the petrous apex [45,46]. The superior petrosal sinus might also be affected, as it runs along its upper border [47]. The inherent anatomy of the internal acoustic meatus constitutes an important landmark that should always be considered in the surgical decision-making process [43,48].

Several pathological lesions can be recognized at the petrous apex and should be considered in the differential diagnosis [46]. The epidermis cysts constitute the most common congenital lesion and usually contain keratinous debris of ectodermal origin and cholesterol [46]. Other common lesions are the cholesteatomas after otitis media, which contain cholesterol crystals, multinucleated giant cells, and hemoglobin metabolism byproducts [46]. Numerous neoplasms, such as chondrosarcomas, chordomas, metastases, and paragon glioma, might also be found in the petrous apex [46]. Petrous apicitis, an infection of the pneumatized bone, PAC, and aneurysms of the petrous segment of the carotid artery are found less frequently in the region [46].

Our patient’s clinical and imaging characteristics could be used to describe the average PAC patient. She was a middle-aged woman, as in the majority of PAC cases. In addition, the brain imaging showed bilateral petrous apex lesions in continuation with Meckel’s cave, which contained clear fluid and an empty sella. All these imaging findings are recognized in more than half of the reported cases. However, the patient did not present any symptoms that could be attributed to the PAC, including trigeminal neuralgia, CSF leak, or altered intracranial pressure [46].

Instead, the patient presented with a TGA. The amnesia attack fulfilled both Caplan’s and Hodges and Warlow's criteria for TGA [4-7]. The episode was witnessed; it involved anterograde amnesia lasting for only a few hours, without any other focal neurological sign or epileptic feature, in the absence of head trauma or loss of personal identity [4]. Furthermore, imaging of the brain failed to identify any ischemic territory that could be associated with the amnesia. However, we did not assess the brain’s electrical activity with EEG since it was an isolated episode of amnesia without epileptic features, requiring no further electrophysiological assessment. The patient did not present cranial nerve palsies, including nystagmus and vertigo, that could be attributed to other neurological conditions [49].

A clear cause-and-effect association between PAC and TGA cannot be established. However, several pathogenetic mechanisms, including mechanical and vascular pathways, could be implicated. Indeed, Mulcahy et al. presented a case where the PAC could be involved in the seizure initiation process, mainly due to its close association with the deep temporal lobe structures [27]. In addition, the mass effect caused by the PAC could compromise, probably temporarily, the blood supply to the adjacent temporal lobe structures [30,36]. According to Ropper et al., both the epileptiform activity and the temporal lobe hypoperfusion theories have been implicated in the pathogenesis of TGA [1].

Alternatively, the PAC might be an “innocent bystander” in a patient suffering from TGA and harboring an empty sella. In other words, the TGA might not be associated with the PAC. The literature showed several cases of PAC that were incidentally identified during the diagnostic workout for other neurological manifestations, particularly headaches [19,22,23,26,30]. In a series of 111 PAC cases reported by Jamjoom and Alorainy, as many as 88 (79.3%) patients were identified incidentally [22].

According to our literature search, the optimal treatment for PAC has yet to be established. As in our current patient, no treatment is warranted in asymptomatic or minimally symptomatic cases. However, in more severe cases, particularly those with CSF leaks and repeated episodes of meningitis, or in cases with documented mass effects, more aggressive management is advocated [17,19,27-30,36,37,41,42]. Once again, the optimal surgical approach is a matter of debate. The literature describes several approaches, but the subtemporal approach to the contents of the middle cranial fossa is the most commonly adopted one [17,27,29,37,41]. Other approaches, such as the retrolabyrinthine [17], the rectosigmoid [17], the endoscopic transnasal approaches [30,32], and the placement of a ventriculoperitoneal shunt (VPS) to control the intracranial pressure [36], were less frequently used.

A major limitation of the current case report is the short follow-up duration. The patient has been advised to seek medical attention in cases of symptom recurrence and has been scheduled for regular follow-up visits. Likewise, the literature review has several important limitations. Firstly, the review is based mostly on low-quality evidence from case reports. Secondly, there is significant heterogeneity in the gathered cases regarding presentation, management, and follow-up. Thirdly, the number of cases with PAC is very small. Therefore, our conclusions could be exposed to significant sources of bias.

## Conclusions

To the best of our knowledge, our case report is the first to show that TGA could occur in association with PAC. However, the nature of the association cannot be clearly established. Therefore, the diagnostic work-up for TGA should include PAC in the differential diagnosis. The literature review showed that patients with PAC are usually middle-aged females, and the presenting symptomatology is diverse, ranging from asymptomatic and incidental cases to severely affected patients with trigeminal neuralgia, epilepsy, and meningitis. Brain CT and MRI show CSF-filled lesions in continuation of Meckel's cave and erosion of the surrounding bony structures. The optimal treatment of PAC has yet to be established, but as our current patient, no further treatment is required in asymptomatic or mildly symptomatic cases. The middle cranial fossa could be reconstructed in patients with CSF leaks and meningitis, mostly through the subtemporal approach. Otherwise, a VPS could be used to moderate the intracranial pressure. The overall prognosis is benign.
